# Stilipedidae Holmes, 1908 (Crustacea, Amphipoda) of the Clarion-Clipperton Zone

**DOI:** 10.3897/zookeys.1274.141416

**Published:** 2026-03-24

**Authors:** Anne Helene S. Tandberg, Lauren E. Hughes

**Affiliations:** 1 University of Bergen, PO box 7800, NO5020 Bergen, Norway University of Bergen Bergen Norway; 2 Senckenberg Research Institute and Museum, Senckenberganlage 25, Frankfurt am Main, Germany Senckenberg Research Institute and Museum Frankfurt am Main Germany; 3 Invertebrates (non-Insects) section, Natural History Museum, London, Cromwell Road, South Kensington, UK Natural History Museum London United Kingdom

**Keywords:** *
Alexandrella
*, *
Astyra
*, deep-sea, North-eastern Pacific, taxonomy

## Abstract

Two new species of the family Stilipedidae from the Clarion-Clipperton Zone in the eastern Pacific are described using integrated taxonomy. The species *Alexandrella haubeni***sp. nov**. and *Astyra mclaughlinae***sp. nov**. are both small and dorsally smooth. The gnathopods of *A. haubeni***sp. nov**. are parachelate and those of *A. mclaughlinae* are subchelate.

## Introduction

**Figure 1.**
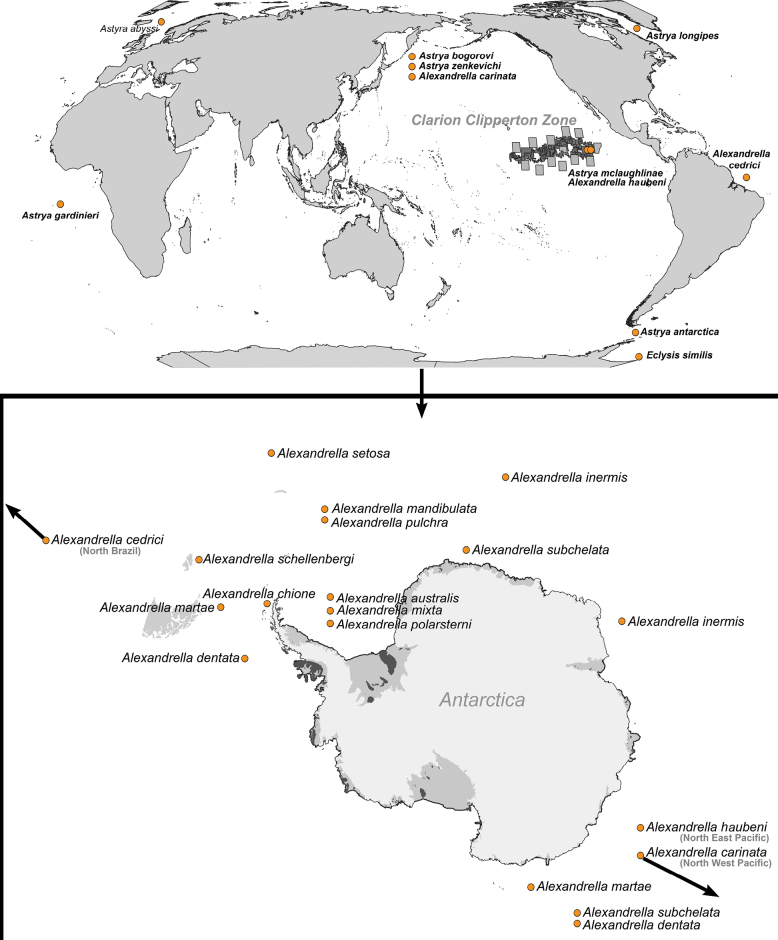
Stilipedidae global distribution **A***Astyra*, *Alexandrella* and *Eclysis* distribution **B***Alexandrella* species distribution. Note that the placement of species in both maps indicate generalised areas and not specific localities.

The Stilipedidae Holmes, 1908 is an infrequently sampled family of deep-sea amphipods. Distributed globally, there are currently 26 species of stilipedids from four genera *Alexandrella* Chevreux, 1911, *Astyra* Boek, 1871, *Eclysis* K.H. Barnard, 1932 and *Stilipes* Holmes, 1908 (Fig. 1). *Alexandrella* (Fig. 1B) is the most specious genus with 14 species, 10 of which are from the (sub)-Antarctic region (Serejo 2014; Souza-Filho et al. 2024).

The family has colonised all oceanic depths being recorded from 230 to 7230 m, the genus *Alexandrella* is among the few taxa with shallow to hadal records. There are just five other genera with such a large depth range, namely *Rhachotropis* S.I. Smith, 1883, *Scina* Prestandrea, 1833, *Byblisoides* K.H. Barnard, 1931, *Liljeborgia* Spence Bate, 1863 and *Scopelocheirus* Spence Bate, 1857 (based on identified and taxonomically described taxa) (Lörz 2010; Lacey et al. 2016; Jamieson and Weston 2023).

At the family level, only 12 of the over 230 described amphipod families have taxonomically described species in shallow to hadal records include Ampeliscidae Krøyer, 1842, Atylidae Lilljeborg, 1865, Eusiridae Stebbing, 1888, Ischyroceridae Stebbing, 1899, Liljeborgidae Stebbing, 1899, Lysianassidae Dana, 1849, Maeridae Krapp-Schickel, 2008, Pardaliscidae Boeck, 1871, Pontogeneiidae Stebbing, 1906, Phoxocephalidae G.O. Sars, 1891, Scinidae Stebbing, 1888 and Scopelocheiridae Lowry & Stoddart, 1997 (Lörz 2010; Lacey et al. 2016; Jamieson and Weston 2023).

The genus *Astyra* with just six described species, is similarly known from an impressive shallow to bathyal depths, 260–4000 m. Collection records are also from extremely disparate locations globally, namely east Greenland, Norway, West Africa, Kamchatka and the Antarctic. The current species is the first North-eastern Pacific, the most tropical and the deepest record in the genus *Astyra*.

Prior to this study there was just one species of Stilipedidae, *Stilipes distinctus* Holmes, 1908, known from the eastern Pacific. Five of the 26 species of Stilipedidae are well collected, being regularly sampled globally in marine surveys in the Northern Hemisphere (*Astyra abyssi* Boeck, 1871 and *S. distinctus* Boek, 1871) and in the Southern Hemisphere (*A. martae* Berge & Vader, 2005, *A. subchelata* Holman & Watling, 1983, *E. similis* K.H. Barnard, 1932) (Berge and Vader 2005) (Fig. 1).

Revisions of the family have been given by Holman and Watling (1983), who included Astyrinae Pirlot, 1934 (previously family Astyridae) as a subfamily of Stilipedidae, and later by Berge and Vader (2005) who contested this classification. d’Udekem d’Acoz and Verheye (2017) examined the Antarctic species of the family Stilipedidae*sensu*Holman and Watling (1983), and found no reason for not including Astyrinae as a subfamily of Stilipedidae; this is the currently accepted taxonomy (Horton et al. 2024). *Alexandrella haubeni* sp. nov. is only the fourth species in the genus recorded outside southern polar waters, while *Astyra mclaughlinae* sp. nov. extends the distribution of the genus to the Pacific Ocean and to greater depths, showing *Astyra* to be a remarkably cosmopolitan genus.

## Materials and methods

The material for the present study was sampled in the central-east Pacific, specifically in the easternmost sector of Clarion-Clipperton Zone (CCZ). The material was collected with epibenthic sledge (EBS) during two scientific deep-sea cruises: the ABYSSLINE-2 (ABYSSal baseLINE project) in 2015 and the MANGAN in 2016. For details of gear deployment and sample processing see Jażdżewska and Horton (2026) and Jażdżewska et al. (2025).

Individuals were initially examined using either a Leica M125 or a Nikon SMZ800 stereomicroscope. The habitus of *Alexandrella haubeni* sp. nov. is presented as a stereomicroscope photograph from a Leica M125, and the habitus for *Astyra mclaughlinae* sp. nov. is presented as a photograph obtained with a confocal laser scanning microscope (CLSM). This holotype was stained in Congo red and acid fuchsin, temporarily mounted onto slides with glycerin and examined with a Leica TCS SPV equipped with a Leica DM5000 B upright microscope and three visible-light lasers (DPSS 10 mW 561 nm; HeNe 10 mW 633 nm; Ar 100 mW 458, 476, 488 and 514 nm), combined with the software LAS AF 2.2.1 (Leica Application Suite, Advanced Fluorescence). A series of photographic stacks were obtained, collecting overlapping optical sections throughout the whole preparation (Michels and Büntzow 2010; Kamanli et al. 2017). The specimens were then dissected and mounted on permanent slides using polyvinyl-lactophenol containing lignin pink. All slides were examined using a Nikon Eclipse Ci compound microscope equipped with a camera lucida. Pencil drawings from the microscope were used as the basis for line drawings. The drawings were inked with Adobe Illustrator CC2024 following the recommendations of Coleman (2003, 2009).

In the figures the following abbreviations were used: A1, 2 = antenna 1, 2; UL = upper lip (labrum); LL = lower lip (labium); Md = mandible; Mx1, 2 = maxilla 1, 2; Mxp = maxilliped; C1–4 = coxa 1–4; G1, 2 = gnathopod 1, 2; P3–7 = pereopod 3–7; Pl1–3 = pleopod 1–3; U1–3 = uropod 1–3; T = telson, UR = urosomite, L = left, R = right.

The registered type material is deposited in the Senckenberg Museum (Frankfurt, Germany) (SMF).

All individuals were subjected to cytochrome *c* oxidase subunit I gene (*COI*) barcoding prior to identification of the species. The molecular procedures are described in Jażdżewska et al. (2025). The sequences were deposited in GenBank with the accession numbers PQ734654 and PQ734740. The Relevant voucher information, taxonomic classifications and sequences are deposited in the data set “DS-AMPHICCZ” in the Barcode of Life Data System (BOLD) (https://doi.org/10.5883/DS-AMPHICCZ) (http://www.boldsystems.org) (Ratnasingham and Hebert 2007). After *COI* sequencing, the BOLD Barcode Index Numbers (BIN) (Ratnasingham and Hebert 2013) were used to genetically identify separate species, and undescribed species in the material were morphologically described.

## Results

### Systematics section

#### Genus Alexandrella

Taxon classificationAnimaliaAllogromiidaMaylisoriidae

Chevreux, 1911

D370F8C0-9506-55BA-BE51-3FABEBF1526C

Alexandrella Chevreux, 1911: 1167—Chevreux 1912: 213; Chevreux 1913: 134; Holman and Watling 1983: 32; J.L. Barnard and Karaman 1991: 703; d’Udekem d’Acoz and Verheye 2017: 166 (see for an extensive synonym list).

##### Generic remarks.

The genus *Alexandrella* is thoroughly treated taxonomically by d’Udekem d’Acoz and Verheye (2017), who synonymised *Bathypanoploea* Shellenberg, 1939 and *Astyroides* Birstein & Vinogradova, 1960 with *Alexandrella* and include four still undescribed Antarctic species of *Alexandrella* in a key to all Antarctic and sub-Antarctic *Alexandrella*. These decisions were based on a combination of morphology and genetic information, and they gave no new formal diagnosis to the genus, but rather they still leant on the diagnosis from Barnard (1969). Recently, Souza-Filho et al. (2024) described a deep-sea *Alexandrella* from northeastern Brazil, and this is the only addition since d’Udekem d’Acoz and Verheye (2017). See Souza-Filho et al. (2024) for a diagnosis of the genus.

#### Alexandrella haubeni sp. nov.

Taxon classificationAnimaliaAllogromiidaMaylisoriidae

0E7519DF-8E81-585D-91CD-43B0302D8879

https://zoobank.org/E73E6186-A005-4931-9BE4-3771186E5DD4

Figs 2, 3, 4, 5

##### Etymology.

Named for Lawrence Hauben, who illustrated amphipods of the North-eastern Pacific off California in the 1960s for Jerry Barnard.

**Figure 2.**
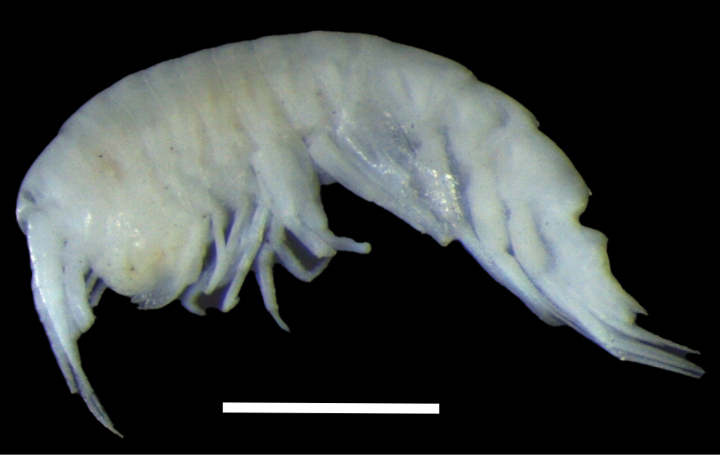
Habitus of *Alexandrella haubeni* sp. nov., SMF 63340. Scale bar: 1 mm.

**Figure 3.**
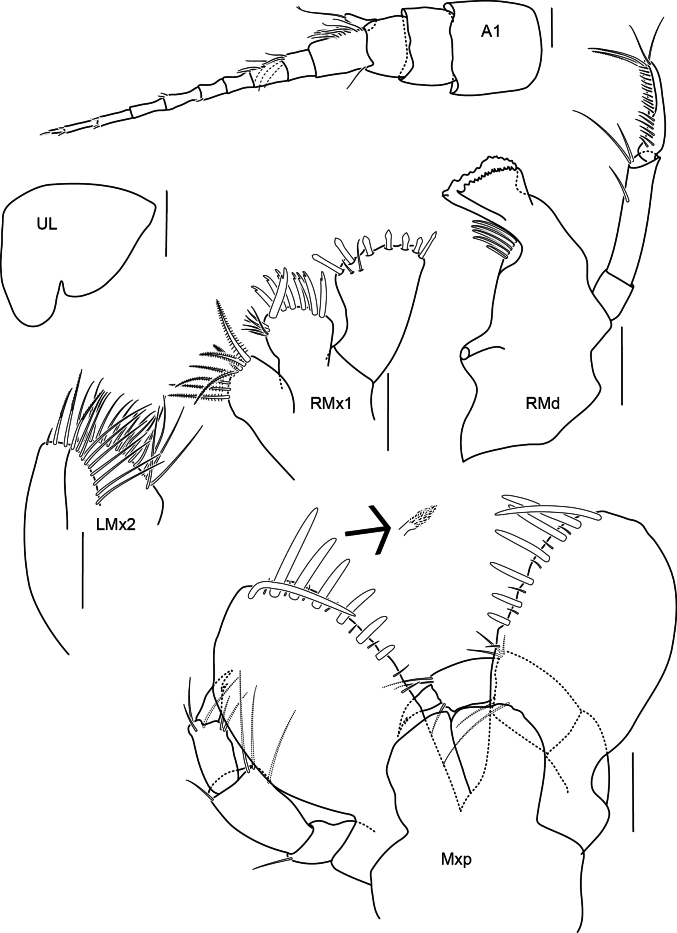
Antenna 1 and mouthparts of *Alexandrella haubeni* sp. nov., SMF 63340 Scale bars: 0.1 mm.

**Figure 4.**
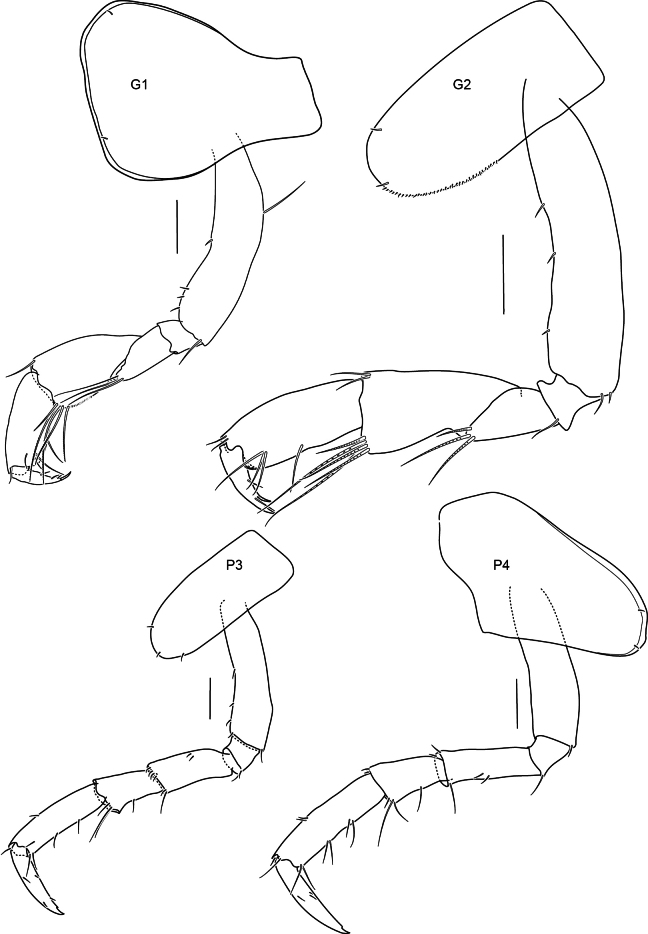
Gnathopods 1–2, pereopods 3–4 of *Alexandrella haubeni* sp. nov., SMF 63340 Scale bars: 0.1 mm.

**Figure 5.**
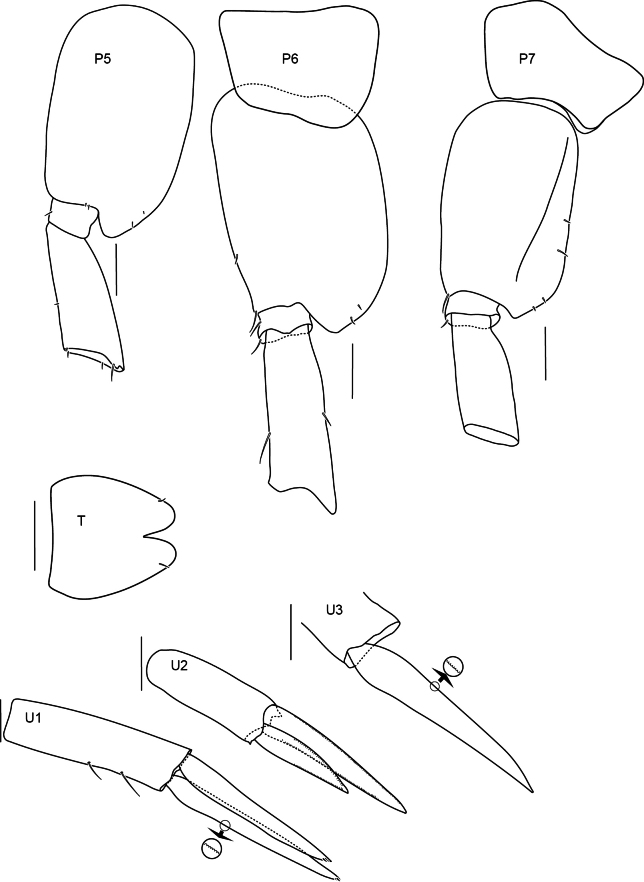
Pereopods 5–7, uropods and telson of *Alexandrella haubeni* sp. nov., SMF 63340 Scale bars: 0.1 mm.

##### Type material.

***Holotype***: Pacific Ocean • SMF 63340; 1 ♂, 3 mm; Clarion-Clipperton Zone; BGR exploration contract area; R/V Kilo Moana, MANGAN 2016, EBS, Ma 16-28, 01/05/2016; 11.828, −117.005; depth 4143 m; COI: PQ734654.

##### Diagnosis.

Labrum asymmetrically lobed. Mandibular incisors broad, irregularly toothed, right lacinia mobilis toothed and not reduced. Gnathopods 1 and 2 parachelate. No dorsal carina or projections, marked dorsal bose on pereonite 1.

##### Description.

Head. Rostrum weakly developed, eyes unknown. Body smooth without dorsal projections. Antenna 1 peduncle article 1 subequal to articles 2 and 3 combined; flagellum with 10 segments; accessory flagellum, 1-articulate, length 3 times breadth, with single apical setae. Antenna 2 (broken). Upper lip as long as broad, asymmetrically lobed, right lobe much larger than left. Mandible well developed, incisors broad, serrate; right lacinia mobilis broad, serrate; accessory setae row with 5 setae; molar present, raised conical, not triturative; palp 3-articulate; article 2 longer than 3, with 2 distal setae; article 3 with marginal setal row and long apical setae. Lower lip lost in dissection. Maxilla 1 inner plate large, lined with 8 plumose setae; outer plate broad, with 8 cuspidate setal teeth; palp 1-articulate (see also other authors illustrations), distally broad, length 1.5 times width, with 7 short, broad robust setae. Maxilla 2 inner plate 1.5 times width of outer plate, margin with long slender simple setae; outer plate margin with long slender plumose setae. Maxilliped inner plate without setae; outer plate broad, margins weakly convex, inner margin lined with 7 short robust setae increasing in length, outer margin lined with minute setules; palp 4-articulate, short, length 0.66 times outer plate, with few setae; dactylus recurved with single medial tooth.

Pereon. Gnathopods 1 and 2 similar, parachelate. Gnathopod 1 coxa anterodistally produced, broadly rounded, coxa partially covering the head; carpus rectilinear, broader than propodus, length 1.8 times breadth; propodus posterior margin with a few long setae; dactylus 0.6 times length of the propodus, weakly recurved with unguis present. Gnathopod 2 coxa anterior and posterior margins straight, ventral margin rounded; carpus rectilinear, broader than propodus, posterodistally produced, length twice breadth; dactylus length 0.8 times propodus, weakly recurved with unguis present. Pereopods 3–4 similar in length. Pereopod 3 coxa margins parallel, ventral margin convex, length 2.5 times width. Pereopod 4 coxa posterior lobe acute. Pereopods 5–7 similar: coxae small as normal for genus, bases posteriorly convex with small acute posterior lobes, merus with parallel margins (all broken above carpus).

Pleon. Pleonites 1–3 without dorsal carina; epimera 1–3 unknown. Urosomite 1 with dorsal bose (a deep excavation medially), without carina. Urosomites 2 and 3 dorsally smooth, without carina. Uropod 1 subequal to uropod 2; biramous, rami subequal. Uropod 2 inner ramus length 0.6 times of outer ramus. Uropod 3 peduncle short, broader than long; rami subequal (one ramus broken in dissection and not drawn). Telson as long as broad, lobes apically convex, cleft 0.25 times length, with pair of subapical setules.

##### Remarks.

*Alexandrella haubeni* sp. nov. is most similar to *Alexandrella subchelata* Holman & Watling, 1983 and *Alexandrella setosa* Serejo, 2014 with both the gnathopods 1 and 2 subchelate to parachelate, as all other *Alexandrella* have the gnathopods 1 and 2 simple. *Alexandrella haubeni* sp. nov. is distinguished by the lack of carina on the dorsal body margin and having only 7 robust setae on the maxilla 1 palp, while both *A. subchelata* and *A. setosa* have carinae present on the pleonites and urosome and more than 20 robust setae on the maxilla 1 palp.

##### Molecular identification.

The species has received a Barcode Index Number from Barcode of Life Data Systems: BOLD:AEA9764 (https://doi.org/10.5883/BOLD:AEA9764).

#### Genus Astyra

Taxon classificationAnimaliaAmphipodaStilipedidae

Boek, 1871

5CA98D46-8D45-5096-B6F6-EEEFA5E0EAF5

Astyra Boeck, 1871: 53—Andres 1997: 81; d’Udekem d’Acoz and Verheye 2017: 178.

##### Generic remarks.

Andres (1997) discussed the taxonomy of the subfamily Astyrinae, and d’Udekem d’Acoz and Verheye (2017) also gave a taxonomic overview of the genus. A diagnosis is found in Barnard and Karaman (1991) and was discussed and updated by Andres (1997). The subfamily Astyrinae (Pirlot, 1934) was previously classified as the family Astyridae (Pirlot 1934; Coleman and Barnard 1991; Berge and Vader 2005) and then as a subfamily of Stilipedidae (Holman and Watling 1987; Barnard and Karaman 1991), which is the curently accepted classification containing the genera *Astyra* and *Eclysis* (Horton et al. 2024).

#### Astyra mclaughlinae sp. nov.

Taxon classificationAnimaliaAmphipodaStilipedidae

3EF56623-8696-5D94-9A9A-BD946B779A92

https://zoobank.org/A675B4C2-5065-4883-AE01-BF14609E20F4

Figs 6, 7, 8, 9

##### Etymology.

Named for Mrs D. McLaughlin who illustrated amphipods of the North-eastern Pacific off California in the 1960s for Jerry Barnard.

**Figure 6.**
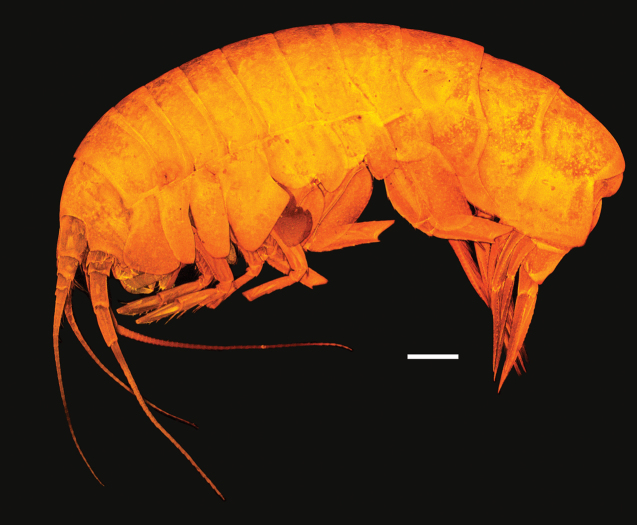
Habitus of *Astyra mclaughlinae* sp. nov., SMF 63341 Scale bar: 1 mm.

**Figure 7.**
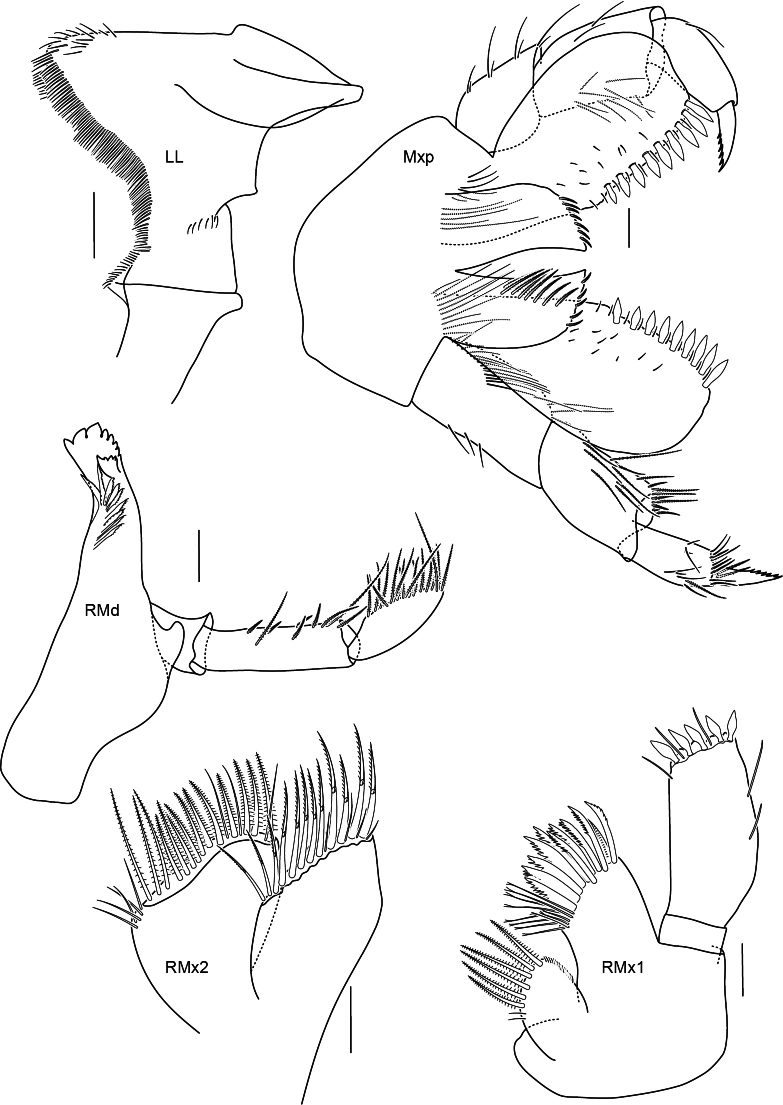
Mouthparts of *Astyra mclaughlinae* sp. nov., SMF 63341 Scale bars: 0.1 mm.

**Figure 8.**
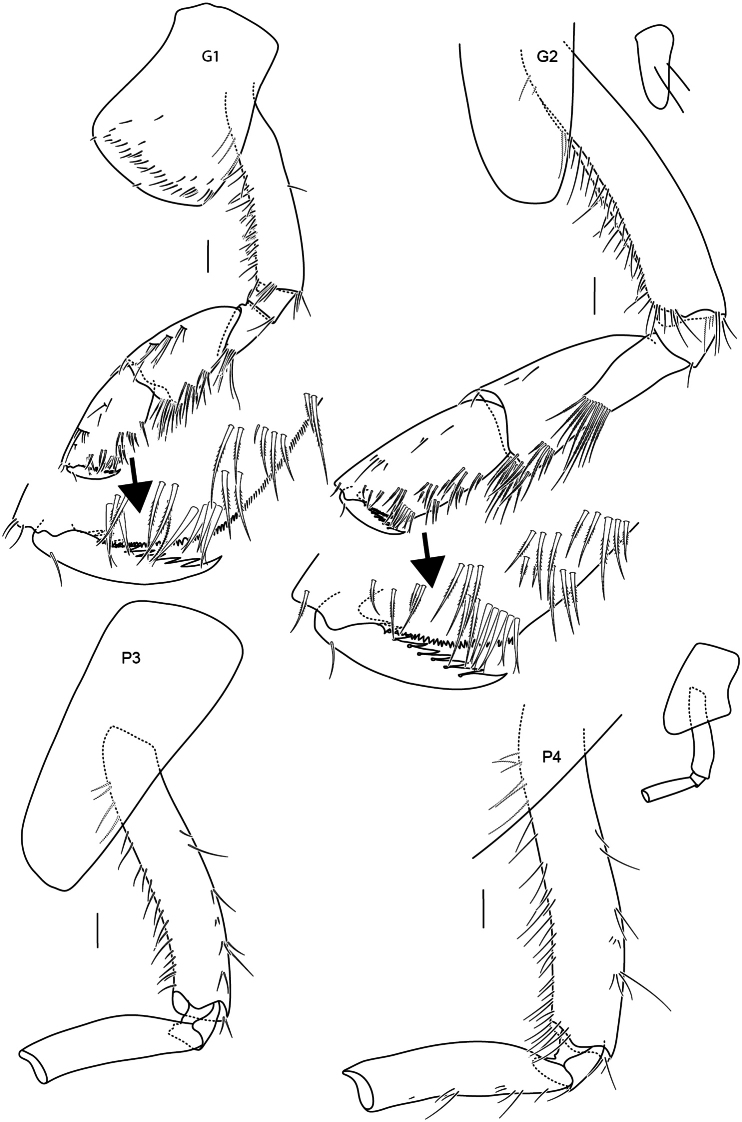
Gnathopods 1–2, pereopods 3–4 of *Astyra mclaughlinae* sp. nov., SMF 63341 Scale bars: 0.1 mm.

**Figure 9.**
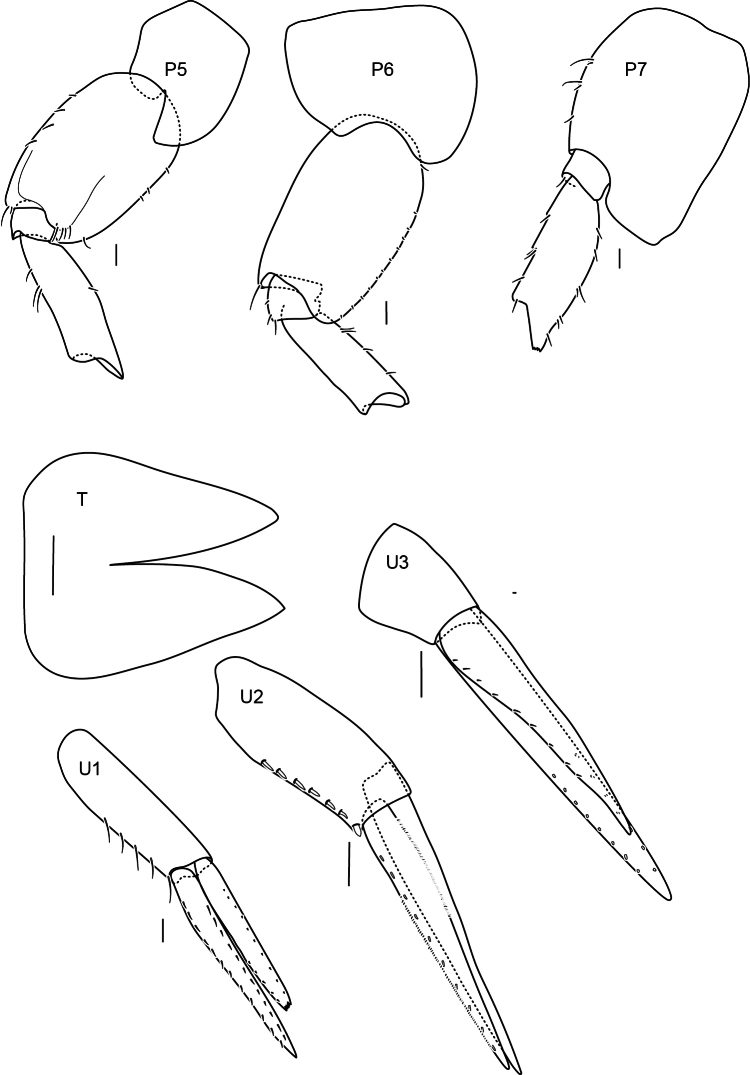
Pereopods 5–7, uropods and telson of *Astyra mclaughlinae* sp. nov., SMF 63341 Scale bars: 0.1 mm.

##### Type material.

***Holotype***: Pacific Ocean • SMF 63341; 1 ♀, 4 mm; Clarion-Clipperton Zone; UKSR-1 exploration contract area, R/V Thompson, ABYSSLINE-2, EBS, AB2-EB01, 18/02/2015; 12.367, −116.55; depth 4209 m; COI: PQ734740.

##### Diagnosis.

Rostrum short, antenna 1 medium length (as long as head and first 5 pereonites combined). Strongly elevated ridge on outer face of bases of P5 and P6 (not on P7). Mandible molar scullcap-like, mandible molar article 2 slightly longer than article 3.

##### Description.

Head shorter than pereonites 1 and 2 together; rostrum weakly developed, lateral cephalic lobe subacute; eyes unknown. Body smooth without projections on dorsal margin. Antenna 1 peduncle article 1 subequal to articles 2 and 3 combined; flagellum with more than 26 articles; accessory flagellum 1-articulate (width not observable). Antenna 2 length subequal to antennae 1, flagellum with more than 49 articles. Upper lip lost in dissection. Mandible well developed, incisors broad, serrate; right lacinia mobilis broad, serrate with 5 serrations, accessory setae row with 9 setae; molar a slightly rised curve (scullcap-like); mandibular palp 3-articulate; article 2 longer than 3, with margin with few setae; article 3 with marginal setal row and long apical setae. Lower lip inner margin sinusoidal, lined with rows of setae. Maxilla 1 inner plate well developed, lined with 6 plumose setae; outer plate broad, with 11 cuspidate setal teeth; palp 2-articulate, distally broad, length 2.1 times width, with 5 short, broad robust setae. Maxilla 2 inner plate 0.7 times width of outer plate, margin with 2 rows of plumose setae; outer plate margin with long slender plumose setae. Maxilliped inner plate with apical and lateral setae; outer plate broad with sparse facial setules, inner margin straight, lined with 9 short robust setae slightly increasing in length distally, outer margin weakly convex and smooth; palp 4-articulate, long, length 1.4 times outer plate, with few setae; dactylus slightly recurved with 5 medial teeth.

Pereon. Gnathopod 1 and 2 similar, subchelate, gnathopod 2 slightly larger than gnathopod 1. Gnathopod 1 coxa anterodistally produced, broadly rounded; carpus subtriangular, broader than propodus, length 1.8 times width; propodus posterior margin with rows of plumose setae, palm subacute, minutely serrate, defined by four robust setae; dactylus length 0.4 times propodus, weakly recurved, unguis present, posterior margin with 8 teeth. Gnathopod 2 coxa anterior margin straight, ventrally rounded; carpus rectilinear, broader than propodus, posterodistally produced, length 1.7 times width; propodus posterior margin with rows of plumose setae, palm subacute, minutely serrate, define by 5 robust setae; dactylus length 0.4 propodus, weakly recurved, unguis present, margin with 8 teeth. Pereopods 3–4 broken. Pereopod 3 coxa margins subparallel, ventral margin convex and slightly prodced ventrally, length 2.2 times width. Pereopod 4 coxa posterior lobe acute. Pereopods 5–6 similar in shape of basis, both with a strong elevated ridge on the outer face of the bases, anterior and posterior margins slightly curved, but paralell, very small posterior lobe. The basis of pereopod 7 does not have an elevated ridge, posterior margin strait, wide and square posterior lobe. (Pereopods 5–7 were broken distally).

Pleon. Pleonites 1–3 without dorsal carina; epimera 1 posteriorly rounded; epimera 2 and 3 posteriorly subquadrate. Urosomite 1–3 without dorsal bose or carina. Uropod 1 subequal to uropod 2; biramous, rami subequal. Uropod 2 biramous, rami subequal. Uropod 3 peduncle short, almost as broad as long; inner ramus length 0.8 times outer ramus. Telson slightly longer than broad, lobes apically subacute, cleft 0.66 times length, without apical setules.

##### Remarks.

*Astyra mclaughlinae* sp.nov. is most smimilar to the cold-water species *Astyra abyssi* Boeck, 1871 (Boreal–North Atlantic) and *Astyra antarctica* Andres, 1997 (Antarctic–Weddell Sea), who both have mandibular palp article 3 slightly shorter than article 2, and the ornamentation of the outer plate of the maxilliped but differs from both these species in the longer maxilliped palp. *Astyra mclaughlinae* is distinguished from *A. antarctica* by its elevated ridges on the bases of pereopods 5 and 6, and from *A. abyssi* by not having an elevated ridge on the basis of pereopod 7. *Astyra mclaughlinae* has a mandibular molar that is close to the molar of *A. antarctica*, also distinguishing it from all other Astyrinae.

##### Molecular identification.

The species has received a Barcode Index Number from Barcode of Life Data Systems: BOLD:AEA9765 (https://doi.org/10.5883/BOLD:AEA9765).

## Discussion

There are six species in *Astyra* according to Horton et al. (2024), and a seventh species, *Astyra longidactyla* Pirlot, 1934, is mentioned both by Barnard and Karaman (1991) and Andres (1997) but seems to have been since forgotten. Including *A. longidactyla*, the genus count of species reaches eight with *Astyra mclaughlinae* sp. nov. Andres (1997) considered the separation of *Astyra* and the monotypic genus *Eclysis* to be “inadequate” without further studies of more Antarctic material, and while stating that the genus *Astyra* needs a thorough re-evaluation, no formal classification changes were suggested. We agree in Andres’ consideration of *Astyra* as a genus in need of taxonomic work, and regret that also we are not able to do that in the scope of the present work. The morphology of the genus is very variable, and the unstable taxonomic history underscores the confusion this taxon has brought to many good amphipodologists.

Two *Astyra* species are described from Kamchatka region, one from each side of the North Atlantic (Davis Strait and the Norwegian Sea), one off West Africa, and one off Elephant Island near the Antarctic Peninsula. *Astyra mclaughlinae* sp. nov. is the third *Astyra* from subtropical waters and the first from the central Pacific Ocean.

Due to the still unsettled state of Astyrinae with the discussion on the separation of *Astyra* and *Eclysis*, we here present only a key to world species of *Alexandrella*—adapted from d’Udekem d’Acoz and Verheye (2017) and Souza-Filho et al. (2024)—and sadly no key to *Astyra*.

### Key to world species of *Alexandrella*

**Table:** 

1	Pereonite 7 with a strong dorsal pointed carina	**2**
–	Pereonite 7 dorsally smooth	**6**
2	Article 1 of peduncle of antenna 1 with short dorsomedial tooth; dactylus of pereopods 3–4 long; posteroventral border of coxa 4 straight or nearly so	**3**
–	Article 1 of peduncle of antenna 1 with long dorsomedial tooth; dactylus of pereopods 3–4 short; posteroventral border of coxa 4 strongly concave	**4**
3	Telson 0.37 cleft	***Alexandrella schellenbergi* (Holman & Watling, 1983)**
–	Telson 0.2 cleft or less	***Alexandrella pulchra* Ren in Ren & Huang, 1991**
4	Anteroventral lobe of head moderately developed, triangular or bluntly triangular; dorsal carina of pleonite 3 with straight profile; posterodistal corner of basis of pereopod 6 broadly rounded	**5**
–	Anteroventral lobe of head very strong, hemi-elliptic; dorsal carina of pleonite 3 with distinctly sigmoid profile; posterodistal corner of basis of pereopod 6 forming a blunt squared angle	***Alexandrella chione* d’Udekem d’Acoz & Verheye, 2017**
5	Pereonite 6 with small posterodorsal carina; crest of urosomite 1 with long posterior tooth with sinous dorsal edge and deep posterior notch	***Alexandrella australis* (Chilton, 1912)**
–	Pereonite 6 without posterodorsal carina (Pereonite 7 with small, triangular and sharp carina; crest of urosomite 1 with small posterior carina with flat dorsal edge and small posterior notch	***Alexandrella carinata* (Birstein & Vinogradov, 1960)**
6	Telson truncate, notched or cleft but not convex	**7**
–	Telson convex and entire (carina of urosomite 1 compact, not elongate)	***Alexandrella polarsterni* (Berge & Vader, 2005)**
7	Gnathopods 1–2 subchelate or parachelate	**8**
–	Gnathopods 1–2 simple	**10**
8	Pleonites 1–2 weak or no dorsal ridge, pleonite 3 may or may not have a dorsal ridge/carina or tooth	**9**
–	Pleonites 1–3 with a dorsal well-developed pointed carina, gnathopods 1–2 densely setose	***Alexandrella setosa* Serejo, 2014**
9	Pleonites 1–2 with a weak dorsal ridge; pleonite 3 with a low sharply toothed carina; gnathopods 1–2 lacking facial seta	***Alexandrella subchelata* Holman & Watling, 1983**
–	Pleonites 1–3 with very weak or no dorsal ridge, urosomite 1 with rounded hump after notch; gnathopods 1–2 with few facial setae	***Alexandrella haubeni* sp. nov**.
10	Pleonites 1–3 strongly toothed; female pereopod 1 with oostegite	**11**
–	Pleonites 1–3 weakly toothed or absent; female pereopod 1 without oostegite	**12**
11	Right mandible with lacinia mobilis reduced to a simple tooth	***Alexandrella mandibulata* Berge & Vader, 2005**
–	Right mandible with lacinia mobilis broad and toothed, but smaller than left one	***Alexandrella martae* Berge and Vader, 2005**
12	Mandibular incisors not toothed along the entire margin; antennae 1–2 subequal in length	**13**
–	Mandibular incisors toothed along the entire margin; antenna 1 shorter than antenna 2	**14**
13	Pereonites 1–7 with a pair of lateral triangular teeth	***Alexandrella dentata* Chevreux, 1912**
–	Pereonites 1–7 smooth	***Alexandrella mixta* Nicholls, 1938 s.l**.
14	Mandible right lacinia mobilis absent; epimeron 3 posteroventral corner rounded; telson entire	***Alexandrella inermis* Bellan-Santini & Ledoyer, 1987**
–	Mandible right lacinia mobilis present and well developed; epimeron 3 postero-ventral corner pointed; telson cleft	***Alexandrella cedrici* Souza-Filho et al., 2024**.

## Supplementary Material

XML Treatment for Alexandrella

XML Treatment for Alexandrella haubeni

XML Treatment for Astyra

XML Treatment for Astyra mclaughlinae

## Additional information

### Author ORCIDs

Anne Helene S. Tandberg https://orcid.org/0000-0003-3470-587X

Lauren E. Hughes https://orcid.org/0000-0002-5679-1732

### Data availability

All of the data that support the findings of this study are available in the main text.
